# Hospital Administration

**Published:** 1897-10-16

**Authors:** 


### The Institutional Workshop.

### HOSPITAL ADMINISTRATION.

### A CHILDREN'S HOSPITAL IN PARIS.

It is a common expression to say that they do such and such a thing better in France. We had occasion to visit the largest children's hospital in Paris a few days ago, and were there afforded evidence that, whatever else may be done better in France, the administration of hospitals, and especially of children's hospitals, is not one of them. Thanks to the ever-increasing sense of responsibility which animates the whole community in this country, so far as children are concerned, each year tends to make the lot of a child in England, whether healthy or ill, happier and better. During fourteen years of our life it was our privilege to reside continuously in a hospital, and from first to last, when oppressed by the burden of anxious work which that residence entailed, we invariably found relief by visiting the children's ward, and seeing the patient courage with which suffering was borne by the little invalids. Thirty years ago it was a much-debated point as to whether or not children did better when placed together in one ward or when scattered promiscuously through the general wards of a great hospital. To-day there is no question as to which of the two systems is the right one. Everybody of experience is convinced that it is unjust to the adult patients to place children in general wards, and that the children themselves are happier and better all together in one ward. Managers know, too, that there is nothing so fruitful in securing supplies as a properly-conducted children's ward in a general hospital. The recognition of these facts has made the lot of a sick child in an English hospital to-day as happy and comfortable as possible.

The French Children's Hospital in Paris was a sad spectacle. The wards were, on the whole, clean and orderly; but the faces of the children in the first ward we entered led us to the conclusion, which was abundantly confirmed in the course of our inspection, that in Paris there is an absence of the loving care and motherly affection which is so marked a feature of English hospitals for sick children. No doubt one chief cause of the unhappy lot of the French child in the Paris hospitals is the absence of efficiently trained nurses. Each ward, of course, had so-called nurses in charge of it, but the stamp of woman employed, her appearance and attitude towards the children, all impressed the trained observer with a feeling that there was an absence of the right kind of control and supervision throughout the hospital. The wards were devoid of every attribute of cheerfulness. There were practically no means of amusement and diversion for the children. It is true that we came across a toy of some kind here and there, but no ward was properly equipped in this respect, and it was evident that the children lack the influence of any intelligent interest taken in their welfare and happiness by the citizens of Paris, and especially by the women of Paris, who might do good work by the exercise of a little self-denial in becoming visitors to the Children's Hospital.

We shall deal later with the question of the nursing in Paris hospitals, and with the controversy which has raged there for some time between the upholders of nuns as opposed to lay nurses in hospitals. The Assistance Publique is organising a system for training lay nurses, male and female, for the hospitals of Paris, but the quality of such service must remain unsatisfactory until those responsible for the selection of the material recognise that they must secure a higher grade of women, workers, who are better educated in every way than those they have so far employed. We gather from an incident which happened in the course of our inspection that the class of women now employed in the Children's Hospital and the Necker Hospital, for instance, and presumably in other Parisian hospitals under the Assistance Publique, is very little, if at all, superior to the Sarah Gamp type, which was the curse of English hospitals forty years ago. At one corner of the grounds attached to the children's hospital a new building is being erected for the accommodation of the nurses. On entering this building we ascertained that it will provide accommodation for female nurses and their husbands, and also for unmarried women. Each married couple is to have two rooms and a kitchen, and the unmarried nurses will be accommodated in rooms containing two beds each. All these nurses will be fed from the hospital kitchen, and it is fair to assume that the husbands and children, as well as the nurses, will be fed at the cost of the ratepayers of Paris. We further observed that the building is so constructed as to give the married and unmarried nurses, and their friends and families, free access to the street when they return to the building from the hospital after doing their work there. Yet this building is the latest type of a nursing home erected in Paris by the chief hospital authority, to whose initiative lal progress must be due, and without such initiative no progress can be made.

It is not our purpose to deal here with the medical treatment of the patients in the hospitals of Paris, but we may mention incidentally that our inspection of the Children's Hospital convinced us that every effort is made by the staff to keep abreast with scientific developments. We wish, however, to confine ourselves entirely to the administrative questions raised by this visit to the Children's Hospital in Paris. We would earnestly appeal to the directors of the Assistance Publique to issue a commission and to appoint trained inspectors to visit some of the principal children's and other hospitals and nurse training schools in Great Britain and the United States. The report of such a commission would place the Assistance Publique in possession of facts of which they are at present apparently ignorant, and so enable them to set about the organisation of a proper system of nursing the hospitals of Paris, which must be fruitful in benefit to the whole community. The right system of nursing the sick, if applied to the Paris hospitals, would immeasurably increase the efficiency of these institutions as houses of cure. It would tend to ameliorate the lot of the patients under treatment, it would bring about the more motherly care of the sick children of Paris, it would increase and entirely change for the better the hygienic condition of the hospitals, and—and this is an important consideration for the ratepayers—it must in the end bring about great economies, a reduction in the expenses now incurred, and other practical benefits. To the inhabitants of Paris, and especially to the ladies of Paris, we would earnestly say—Will you not rise to your responsibilities in regard to the poor sick children of the beautiful city it is your privilege to dwell in? Will you not take the children's hospitals, as a beginning, under your care, and at any rate devote sufficient time to pay a visit to at least one such hospital in order that you may realise how much of human suffering is borne there, and how much happiness your occasional presence and ripe experience must bring to the small inmates, who at present suffer in solitude and without adequate means of entertainment or diversion. We are quite willing to believe that the present condition of affairs is due to a want of thought rather than to a want of heart on the part of the women of Paris. We hope, however, that this article may lead to the formation of one or more committees of women who will pledge themselves to secure that the sick children in Paris hospitals shall for the future be at least as comfortable, as well cared for, and as happy as those in other countries, where the women have made the children's hospital their special charge and domain. In any case, we can assure Parisians that the feeling of depression due to a knowledge of the present state of affairs in the large children's hospitals in Paris, compared with what it might be and ought to be, has remained with us from the hour we left the institution. We are further impelled to say with assured certainty that, if only a few good women in Parisian society will take this matter up and visit every children's hospital, the necessary changes will be brought about without hesitation or delay. Personal service, such as that needed by the poor sick children of Paris, from Parisian ladies, when cheerfully rendered, is of infinitely greater value than any contribution of money, however large the amount.

